# The complete chloroplast genome of *Arcangelisia gusanlung* H.S.Lo (Menispermaceae), an important traditional medicine from South China

**DOI:** 10.1080/23802359.2021.1993099

**Published:** 2021-10-23

**Authors:** Guihua Wen, Sheng Chen, Lingling Fan, Lei Zeng, Bingqiang Xu

**Affiliations:** a Key Laboratory of Plant Resources Conservation and Sustainable Utilization, South China Botanical Garden, Chinese Academy of Sciences, Guangzhou, China; b College of Life Sciences, University of Chinese Academy of Sciences, Beijing, China; c Guizhou Institute of Biology, Guiyang, China; d Key Laboratory of Forest Cultivation, Conservation and Utilization, Guangdong Academy of Forestry, Guangzhou, China

**Keywords:** Complete plastid genome, *Arcangelisia gusanlung*, phylogenetic relationship

## Abstract

*Arcangelisia gusanlung* H.S.Lo is widely used as a folk medicine by the Dai and Li peoples. Here, we report the first complete chloroplast (cp) genome sequence for this species based on Illumina paired-end sequencing data. The cp genome was 162,509 bp in length with a small single-copy (SSC) region of 20,852 bp, a large single-copy (LSC) region of 91,449 bp, and two separated inverted region of 25,104 bp. In total, 129 unique genes were identified of this genome, including 84 protein-coding genes, 37 tRNA genes, and eight rRNA genes. The GC contents of this genome is 37.8%. Phylogenetic analysis based on 13 complete cp genomes showed a strong sister relationship with *Tinospora cordifolia* (Willd.) Miers and *Tinospora sinensis* (Lour.) Merr. This complete genome of *A. gusanlung* will provide valuable information to elucidate the mechanism of speciation of *Arcangelisia* Becc.

*Arcangelisia* Becc. is a small genus in Menispermaceae, comprised of only four species; *Arcangelisia gusanlung* H.S.Lo is the only recorded plant of the genus in China (Luo et al. 2008). This species has large woody vines and is widely distributed in the south of China including the provinces of Hainan, Guangdong, and Guangxi. The stems and roots of this plants are used in Hainan as a traditional antipyretic medicine much like the traditional Chinese medicinal plant *Phellodendron chinense* Schneid. (Yu et al. 2014). Pharmacological research shows that *A. gusanlung* has anti-inflammatory, analgesia, antipyresis, antitussive, expectorant, and antidiarrhea effects (Hu et al. 2013) that maybe associated with the 15 protoberberine-type alkaloids obtained from this plant. Belonging to a isoquinoline alkaloid class, protoberberine alkaloids encompass a diverse class of secondary metabolites with many pharmacologically active members, such as berberine and palmatine (Yu et al. 2014). However, until now, genomic data for this important Chinese folk medicine are scarce. Here, we report the first complete chloroplast (cp) genome sequence for *A. gusanlung* and verified its phylogenetic position in Menispermaceae.

The fresh leaves of *A. gusanlung* were collected from the South China Botanical Garden, Chinese Academy of Sciences (CAS) (113.373785 E; 23.187841 N; Wen & Cheng GSL01). The voucher specimen (accession number: IBSC819035) was deposited at the herbarium of South China Botanical Garden (Fei-Yan Zeng, zengfeiy@scib.ac.cn). The total genomic DNA was extracted from the silica-gel dried leaves following the modified Tiangen Kit (Tiangen Biotech, Beijing, China). The extracted DNA was stored in the Plant Resources Conservation and Sustainable Utilization Laboratory in South China Botanical Garden. Then, the genomic library (paired-end, PE = 150 bp) was sequenced on an Illumina HiSeq X Ten platform at Beijing Genomics Institute (Shenzhen, China). The clean data were quality-controlled by using SOAPnuke 2.X (Chen et al. 2018). In total, 17,109,640 reads (2,566,446,000 bp) were obtained and used to assemble the cp genome after filtering and trimming the low-quality reads. The complete cp genome assembly was executed on NOVOPlasty 2.6.3 (Dierckxsens et al. 2017) with the default k-mer of 39-59. The cp genome of *Tinospora cordifolia* (Willd.) Miers (GenBank accession NC_042153) was used as the reference for assembling. Genes were annotated using GeSeq and visually checked in Geneious version 11.0.3 (Kearse et al. 2012; Tillich et al. 2017). Finally, the complete annotated cp genome was submitted to GenBank (accession MW829779).

The complete cp genome of *A. gusanlung* was 162,509 bp in length with the typical quadripartite structure of angiosperms, including a small single-copy (SSC) region of 20,852 bp, a large single-copy (LSC) region of 91,449 bp, and a pair of inverted repeats (IRs) of 25,104 bp. The genome contained 129 genes, including 37 tRNA genes, eight rRNA genes, and 84 protein-coding genes. The overall GC content in the cp genome of *A. gusanlung* was 37.80%, the corresponding values for the SSC, LSC, and IR regions were 32.60, 35.90, and 43.50%, respectively.

To identify the phylogenetic position of *A. gusanlung* in Menispermaceae, the cp genome sequences of *A. gusanlung*, 10 other species of Menispermaceae and two outgroups (*Berberis amurensis* Rupr. and *Nandina domestica* Thunb.) were aligned using MAFFT 7.0 (Katoh and Standley 2013). A maximum-likelihood (ML) tree was constructed by using IQ-TREE (Nguyen et al. 2015) based on the best model of GTR + F+R2 and 1000 bootstrap replicates (Figure 1). The phylogenetic tree indicates that *A. gusanlung* forms a well supported cade with two species of *Tinospora*. This *Arcangelisia*+*Tinospora* clade is sister to a well supported clade containing all other Menispermaceae species for which cp genome sequence data are publicly available. The plastome data presented here provides a fundamental genomic resource for future conservation and pharmacological studies of *A. gusanlung* as well as phylogenetic relationships within Menispermaceae.

**Figure 1.**
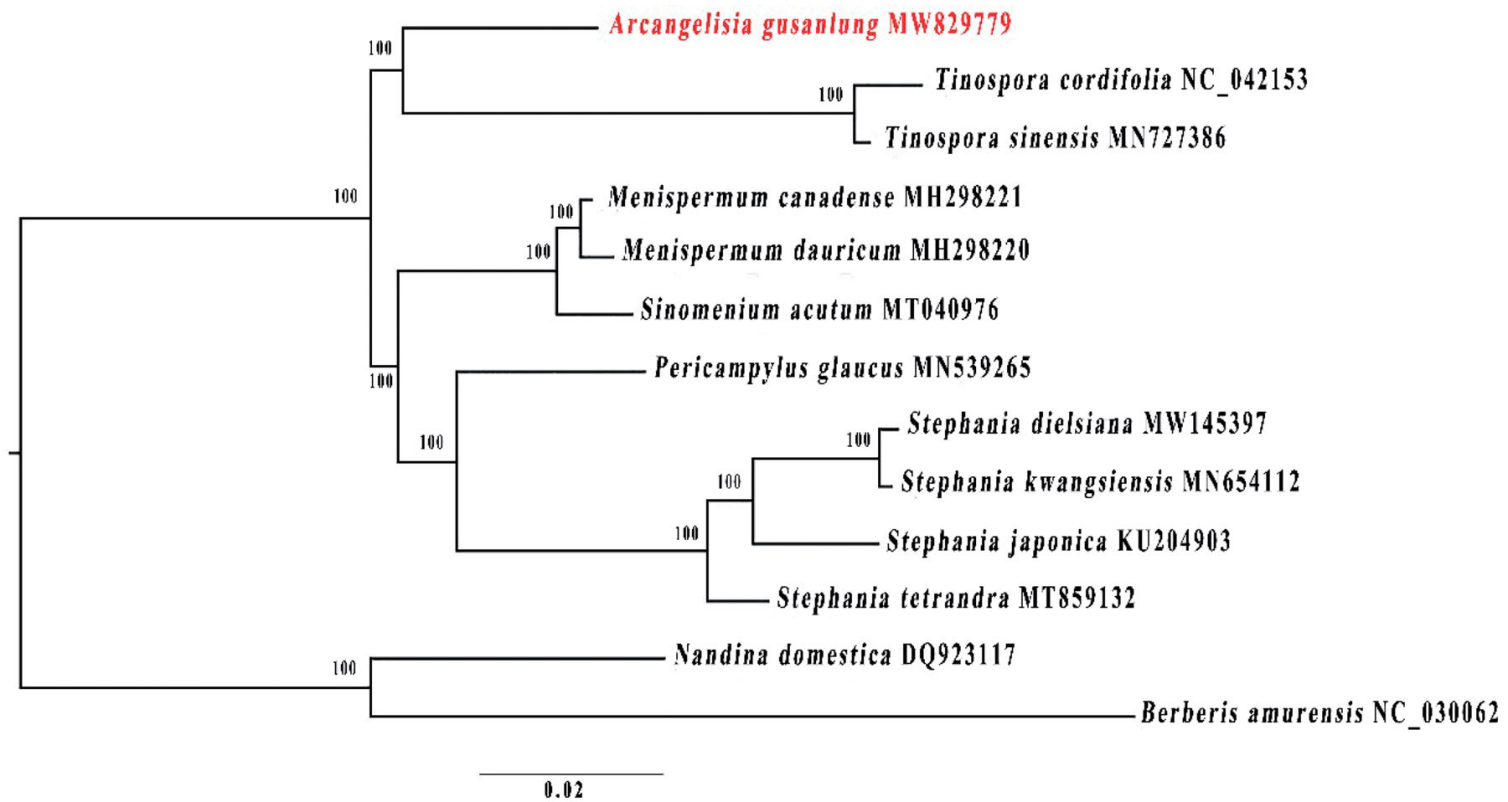
Maximum-likelihood tree based on 13 complete chloroplast genomes of Menispermaceae. Bootstrap support values are shown at the branches. *Berberis amurensis* and *Nandina domestica* were set as outgroups.

## Data Availability

The genome sequence data that support the findings of this study are openly available in GenBank of NCBI (https://www.ncbi.nlm.nih.gov/) under the accession no. MW829779. The associated BioProject, SRA, and Bio-Sample numbers are PRJNA758265, SRR15662106, and SAMN21019820, respectively.
